# A multi-cantilever beam low-frequency FBG acceleration sensor

**DOI:** 10.1038/s41598-021-98055-z

**Published:** 2021-09-16

**Authors:** Li Hong, Rui Sun, Zhongchao Qiu, Zhiming Han, Yanan Li

**Affiliations:** 1 Institute of Disaster Prevention, School of Electronic Science and Control Engineering, Sanhe, 065201 Hebei China; 2 Key Laboratory of Seismic Dynamics of Hebei Province, Sanhe, 065201 Hebei China; 3 Institute of Geophysics, China Earthquake Administration, Beijing, 100081 China

**Keywords:** Natural hazards, Optics and photonics, Mechanical engineering

## Abstract

The acquisition of 2–50 Hz low-frequency vibration signals is of great significance for the monitoring researches on engineering seismology, bridges & dams, oil & gas exploration, etc. A multi-cantilever beam low-frequency FBG acceleration sensor is proposed against the low sensitivity that predominates in the low-frequency vibration measurement by FBG acceleration sensors. Structural parameters of the sensor is subjected to simulation analysis and optimization design using the ANSYS software; the real sensor is developed based on the simulation results in the following manner: Three rectangular of the cantilever beams are evenly arranged around the mass block at 120°to improve the sensitivity and alleviate the transverse crosstalk of sensor; in the end, a performance test is performed on the sensor. According to the research findings, the sensor, whose natural frequency is approximately 64 Hz, is applicable for monitoring the low-frequency vibration signals within the range 16–54 Hz. The sensor sensitivity is approximately $87.955\,{\text{pm/m}}\;{\text{s}}^{ - 2}$, the linearity being greater than 99%, the transverse interference immunity being lower than 2.58%, and the dynamic range being up to 86 dB. The findings offer a reference for developing sensor of the same type and further improving the sensitivity of fiber optic acceleration sensor.

## Introduction

The acquisition of 2–50 Hz low-frequency vibration signals is of great significance for the monitoring researches on engineering seismology, bridges & dams, oil & gas exploration, etc^1,2^. In recent years, acceleration sensors, one of the key devices for dynamic acquisition of vibration signals, has been extensively used for the monitoring of large structures and environmental safety^3,4^. By contrast, traditional electronic accelerometers have limited applications due to their susceptibility to electromagnetic interference. Fiber Bragg Grating (FBG) is an optical sensing element featuring small footprint, strong stability, excellent electromagnetic immunity, high measurement accuracy, etc. Such merits enable FBG to replace electronic accelerometers in seismic wave detection, large structure health monitoring, oil & gas exploration, etc., thereby improving the remote online monitoring capabilities in harsh environments^5–7^.

In recent years, low-frequency FBG acceleration sensors have been extensively and intensively studied at home and abroad^8,9^. Dingyi Feng et al.^10^ proposed a dual-cantilever beam compact FBG acceleration sensor, where two identical L-shaped rigid cantilever beams are connected back-to-back and fixed as mass blocks at the free end of the rectangular cantilever beam. That structure offers a dynamic range of 3 G, but it's less sensitive. M.M.Khan et al.^11^ proposed a FBG accelerometer with L-shaped non-uniform cross-section cantilever beams, where dual FBGs were used for sensor temperature self-compensation; the sensor sensitivity was $306{\text{ pm/g}}$ at a resonant frequency above 150 Hz; however, the sensor sensitivity was restricted to $40{\text{ pm/g}}$ within the frequency range below 50 Hz. Qinpeng Liu^12^ designed a dual-cantilever beam FBG accelerometer based on the theory of coupled film, and it exhibited favorable linear response within the frequency range 4–30 Hz, with the corresponding range of sensitivity being $7.76 \, - 10.8{\text{ pm/m}}\;{\text{s}}^{ - 2}$. Om Prakash et al.^13^ presented a FBG accelerometer with dual L-shaped cantilever beams, where two FBGs were integrated in a differential sensing device to broaden the operational range in addition to improving the sensitivity. Despite the fruitful results that have been achieved in low-frequency FBG acceleration sensors in recent years, low sensitivity has been a bottleneck hindering the practical engineering application of low-frequency FBG acceleration sensors.

Aiming at the problem that the sensitivity of the current FBG acceleration sensor is generally low during the measurement of low-frequency vibration signals, the paper proposes a multi-cantilever beam low-frequency FBG acceleration sensor. Three rectangular cantilever beams are evenly arranged around the mass block at 120° to improve the sensitivity, in addition to alleviating the transverse crosstalk of sensor. Furthermore, the two-point attachment technique was adopted by attaching the FBG along the cantilever beam centerline between the hold-down support and mass block; a pre-stress is applied to avoid FBG chirp and enhance the sensor sensitivity^14,15^. The multi-cantilever beam FBG acceleration sensor was theoretically analyzed in terms of sensitivity, natural frequency, transverse interference immunity, etc.; simulation analysis and optimization design were performed for the sensor using the ANSYS software; the real sensor was fabricated based on the simulation result, and its performance was tested through experiments.

## Structural design and theoretical analysis of the sensor

### Structural design of the sensor

**Figure 1:**
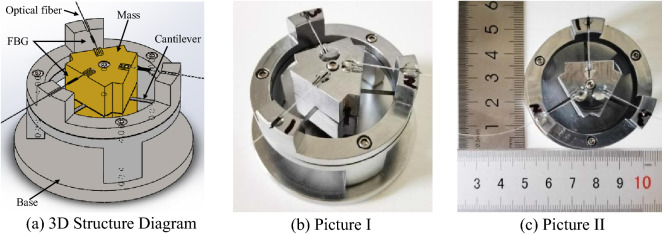
Structure diagram of the sensor [**(a)** was generated by Solidworks 2016 × 64 (https://www.solidworks.com/); **(b,c)** were shot by Huawei P40pro].

The multi-cantilever beam low-frequency FBG acceleration sensor was principally composed of three rectangular cantilever beams, two mass blocks, the hold-down support, and three FBGs with similar central wavelengths. The rectangular cantilever beam was fabricated at 120° intervals on a circular spring steel sheet, and the two mass blocks were fixed through screws on the front and back sides of the cantilever beam; then, the beams and mass blocks were integrally fixed onto the hold-down support. The FBG was fixed into the grooves of the mass block and hold-down support along the cantilever beam centerline through two-point attachment, and was then pre-stressed; the structure is detailed in Fig. 1.

With FBG as its sensing element, the multi-cantilever beam low-frequency FBG acceleration sensor converts mechanical vibration signals into FBG central wavelength shift signals. When the sensor is excited by the vibration signal in the Z direction, the mass block drives the free end of cantilever beam to move up and down under the action of inertial force, which in turn causes the FBG to stretch or compress; as a result, the central wavelength of the FBG reflected wave would shift. Therefore, the acceleration value of the external vibration signal can be determined by simply measuring the offset of the FBG center wavelength.

### Theoretical analysis of the sensor

**Figure 2:**
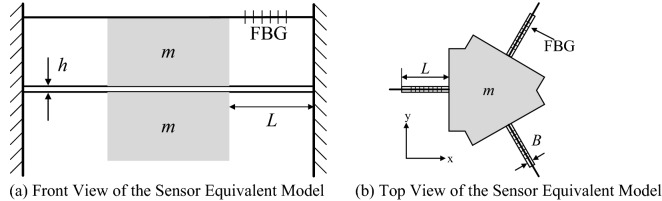
Structural equivalent model of the sensor [**(a,b)** were generated by Microsoft Visio Professional 2016 (https://www.microsoft.com/zh-CN/download/details.aspx?id=51188)].

The structural equivalent model of the sensor is shown in Fig. 2, where *h* represents the thickness of cantilever beam; *L* is the length of cantilever beam; *B* denotes its width; *m* stands for the mass of the sensor mass block.

The mechanical structural portion of the multi-cantilever beam FBG acceleration sensor can be simplified to a second-order single- Degree-Of-Freedom (DOF) forced vibration system consisting of *m* (mass block), k (equivalent spring stiffness of the cantilever beam) and $\gamma$(damping). In the coordinate system shown in Fig. 2b, the kinematic equation of the mass block is

1 $$M\frac{{d^{2} y}}{{dx^{2} }} + \gamma \frac{dy}{{dx}} + ky = - ma_{g}$$

where, *m* represents the mass of the mass block; *y* means the absolute displacement of mass block;$\gamma$ denotes the damping constant of vibration system; *k* is the equivalent elastic coefficient of the vibration system; *a*_*g*_ stands for the acceleration of the vibration signal received by the sensor.

When the forced vibration of the mass block causes the cantilever beam to deform, the resulting strain *ε* in the cantilever beam should be

2 $$\varepsilon { = }\frac{2FL}{{EBh^{2} }}$$

where, *F* is the inertial force on the mass block; *L* denotes the length of cantilever beam; *E* represents the elasticity modulus of the cantilever beam material; *B* means the width of cantilever beam; *h* stands for the thickness of cantilever beam.

FBG refers to an intrinsic optical sensor where the refractive index of the fiber core changes periodically. The region where the refractive index of fiber core changes periodically is equivalent to a periodic vertical mirror, where incident light produces reflected light that interferes with each other, which causes the reflected spectrum to appear as a peak. The stretching or compression of FBG will change the period of the refractive index of fiber core, which causes the reflection peak to shift. Therefore, the FBG attached between the hold-down support and the mass block will be longitudinally deformed, which in turn causes the central wavelength to change in the following magnitude

3 $$\Delta \lambda { = }\lambda (1 - P_{e} )\varepsilon$$

where,$\lambda$ represents the central wavelength of FBG;$P_{e} \approx 0.22$ denotes the elasto-optical coefficient;$\varepsilon$ stands for the strain in FBG;$\Delta \lambda$ is the FBG wavelength shift caused by strain.

Since the cantilever beam and FBG share the same plane, the deformation of cantilever beam can be approximated as that of the FBG. The following is true when Eq. (2) is substituted into Eq. (3)

4 $$\Delta \lambda { = }\lambda (1 - P_{e} )\frac{2FL}{{EBh^{2} }}$$

To attain low-frequency vibration signals in a more favorable manner, the sensor should be favorably sensitive within a certain frequency band. Equation (4),$F = ma_{g}$ and $S{ = }{{\Delta \lambda } \mathord{\left/ {\vphantom {{\Delta \lambda } {a_{g} }}} \right. \kern-\nulldelimiterspace} {a_{g} }}$ are simultaneously established to identify the following sensitivity of acceleration sensor

5 $$S{ = }\lambda (1 - P_{e} )\frac{2ML}{{EBh^{2} }}$$

According to Eq. (5), the sensor sensitivity is associated with the strain generated when the sensor's sensing element is forced to vibrate. The sensitivity referred to in this paper is peak-to-peak, i.e., 2*S*.

The natural frequency of FBG acceleration sensor determines the input signal frequencies in all measurements. When it comes to seismic wave detection, large structure health monitoring, oil & gas exploration, etc., vibration signals with frequencies below 50 Hz and low amplitudes are often critical. The natural frequency of sensor is

6 $$F_{0} = {{\sqrt {\frac{{EBh^{3} }}{{2ML^{3} }}} } \mathord{\left/ {\vphantom {{\sqrt {\frac{{EBh^{3} }}{{2ML^{3} }}} } {2\pi }}} \right. \kern-\nulldelimiterspace} {2\pi }}$$

## Structural parameter analysis and simulation

### Effect of structural parameters on sensor capabilities

The vibration performance and measuring range of sensor are associated with the natural frequency of sensor. To achieve higher sensitivities within the vibration frequency range of 2–50 Hz, it's essential to maximize the sensor sensitivity $S$ through optimized design, and to ensure appropriate values are available for natural frequency $F_{0}$, so as to guarantee higher sensitivity indexes within the favorable frequency response range. According to the outcome of theoretical analysis, the length (*L*), width (*B*), and height (*h*) of the cantilever beam are key parameters affecting sensor sensitivity $S$ and natural frequency $F_{0}$. Hence, with the other parameters fixed, the cantilever beams in various sizes were modeled with Solidworks and subjected to simulation analysis with ANSYS to study the effects of these three key parameters on sensor sensitivity and natural frequency, respectively.

**Figure 3:**
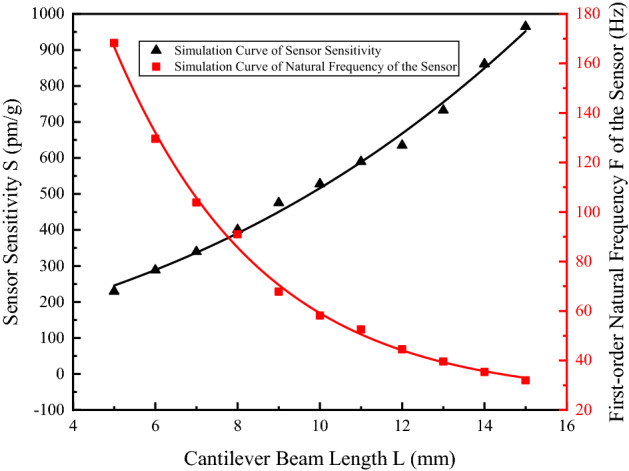
Relation curve of the variation of $S$ and $F_{0}$ with $L$ [the figure was generated by Origin 2016 × 64 (https://www.originlab.com/2016)].

First of all, since the probe should be as small as possible to facilitate the actual installation and operation, the effects of length *L* of the cantilever beam that varied within the range 5–15 mm on sensor sensitivity $S$ and natural frequency $F_{0}$ were discussed; the simulation result and its fit curve is shown in Fig. 3.

As shown in Fig. 3, sensor sensitivity $S$ increased with the increase in length $L$ of the cantilever beam; natural frequency $F_{0}$ increased with the decrease in length $L$ of the cantilever beam. Where 5 mm < $L$ < 10 mm, natural frequency $F_{0}$ decreased sharply with the increase in $L$; where $L$ > 10 mm, the variation leveled off progressively. In view of the fact that the sensor was designed to acquire low-frequency vibration signals below 50 Hz, the natural frequency $F_{0}$ of sensor was chosen to be greater than 50 Hz. Besides, since sensor sensitivity $S$ should not be excessively low, the length of cantilever beam is expected to fall within the range 8 mm < $L$ < 10 mm.

**Figure 4:**
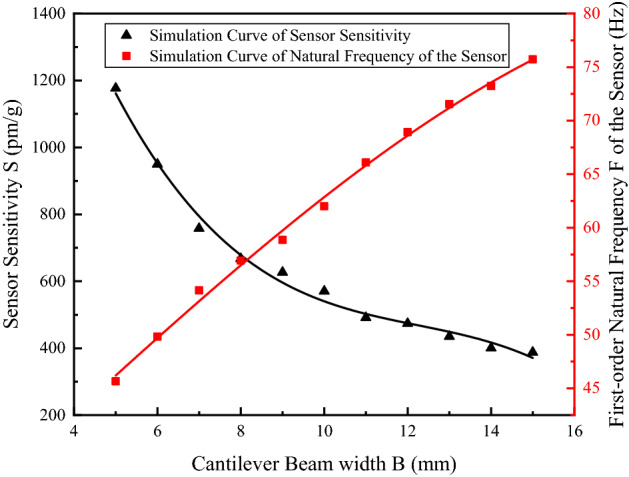
Relation curve of the variation of $S$ and $F_{0}$ with $B$ [the figure was generated by Origin 2016 × 64 (https://www.originlab.com/2016)].

Secondly, analyze the influence of the cantilever beam width *B* on the sensitivity $S$ and natural frequency $F_{0}$ of the sensor when the width of the cantilever is changed from 5 to 15 mm. The simulation result and its fit curve are shown in Fig. 4.

As shown in Fig. 4, sensor sensitivity $S$ decreased with the increase in width $B$ of the cantilever beam; natural frequency $F_{0}$ increased with the increase in width $B$ of the cantilever beam. When $B$ ≤ 9 mm, the $S$ curve exhibited a clear downward trend with the increase in $B$; the change leveled off when 10 mm < $B$ < 14 mm. To achieve a higher sensitivity, the cantilever beam width *B* should be minimized; in consideration of the restrictions on material selection and actual processing conditions of cantilever beam,$B$ is recommended to be 12 mm.

**Figure 5:**
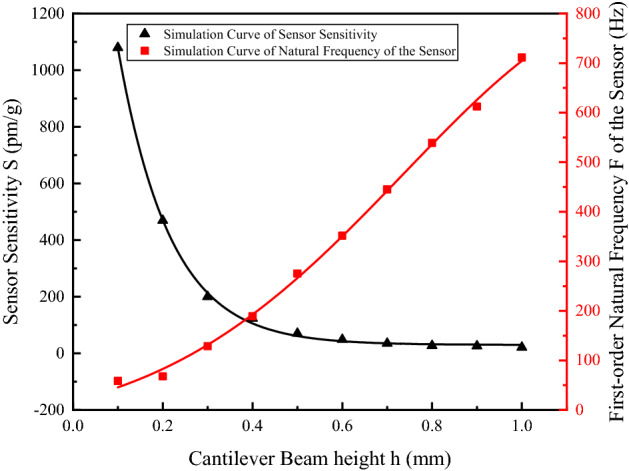
Relation curve of the variation of $S$ and $F_{0}$ with $h$ [the figure was generated by Origin 2016 × 64 (https://www.originlab.com/2016)].

Finally, the effect of variation of cantilever beam height $h$ between 0.1and 1 mm on sensor sensitivity $S$ and natural frequency $F_{0}$ was considered. The simulation result and its fit curve are shown in Fig. 5.

As shown in Fig. 5, sensor sensitivity $S$ decreased with the increase in height $h$ of the cantilever beam; natural frequency $F_{0}$ increased with the increase in height $h$ of the cantilever beam. To address the sensor requirements for sensitivity and natural frequency,$h$ is recommended to be 0.2 mm.

Through the analysis of the effects of three key parameters on the sensor sensitivity and natural frequency with consideration to the material selection and processing conditions, spring steel (65Mn, Young modulus $E = 210$ GPa; Poisson's ratio $\mu = 0.28$); length of each cantilever beam *L* = 9 mm; width $B$ = 1.2 mm; thickness *h* = 0.2 mm was taken as the cantilever beam material used in production of sensors. Due to the constraints of sensor size and processing cost, the mass block was required to be heavy in small size; hence, brass (H62), which is denser, was chosen as the material of mass block; the calculated mass of mass block was approximately 0.042 kg.

### Static stress analysis of sensor structure

**Figure 6:**
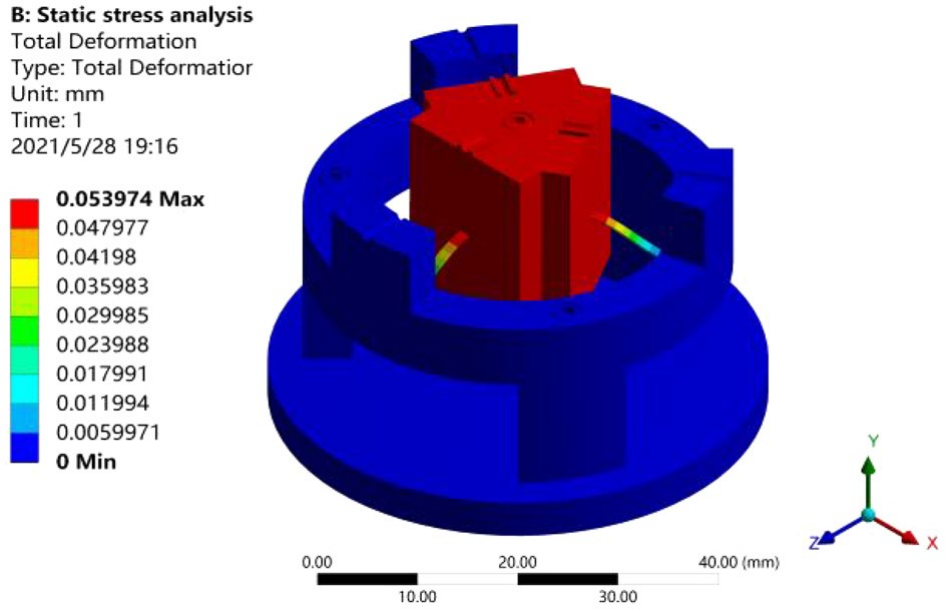
Static stress simulation analysis [the figure was generated by ANSYS workbench 19.0 (https://www.ansys.com/zh-cn/products/structures/ansys-mechanical)].

The sensor was modeled with Solidworks based on the structural parameters identified through the above-noted analysis, and the result was imported into the ANSYS software for simulation analysis. First, fixed constraint was applied to the bottom of sensor model; the connection surfaces between cantilever beam and mass block and between cantilever beam and hold-down support were designated as fully bound support constraints; an external load with standard earth's gravitational acceleration $g$ was applied to the sensor as a whole; the model was subjected to static stress simulation analysis through meshing to draw the strain plot of model, as shown in Fig. 6.

As shown in Fig. 6, the maximum deformation was observed at the free end of sensor, and the deformation decreased toward the fixed end; the maximum deformation at the free end was approximately 0.054 mm.

### Modal analysis of the sensor structure

**Figure 7:**
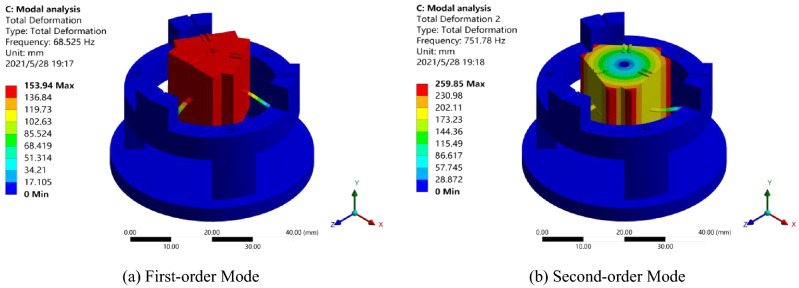
Modal analysis [**(a,b)** were generated by ANSYS workbench 19.0 (https://www.ansys.com/zh-cn/products/structures/ansys-mechanical)].

Mode is an inherent property of the sensor structure itself; the sensor model was subjected to modal analysis based on the result of static stress analysis on sensor. The natural frequencies of the first-order, second-order, third-order, and fourth-order modes identified were 68.5 Hz, 751.8 Hz, 2052.7 Hz, 2053.8 Hz, respectively; the main mode graph extracted from the modes of the first two orders is shown in Fig. 7.

As shown in Fig. 7a, the first-order mode is simple harmonic vibration, indicating that the sensor model vibrates along the Y-axis under external excitation. As shown in Fig. 7b, the second-order mode is a rotational mode, indicating that the sensor model rotates around the Y-axis under external excitation. By comparing the frequencies of the modals of each order, it can be known that, the frequency of the first-order modal was quite different from the frequencies of the second-order, third-order, and fourth-order modals, it indicates that the sensor in this structure is designed with small cross coupling that helps reduce cross interference.

### Experimental test of the sensor

**Figure 8:**
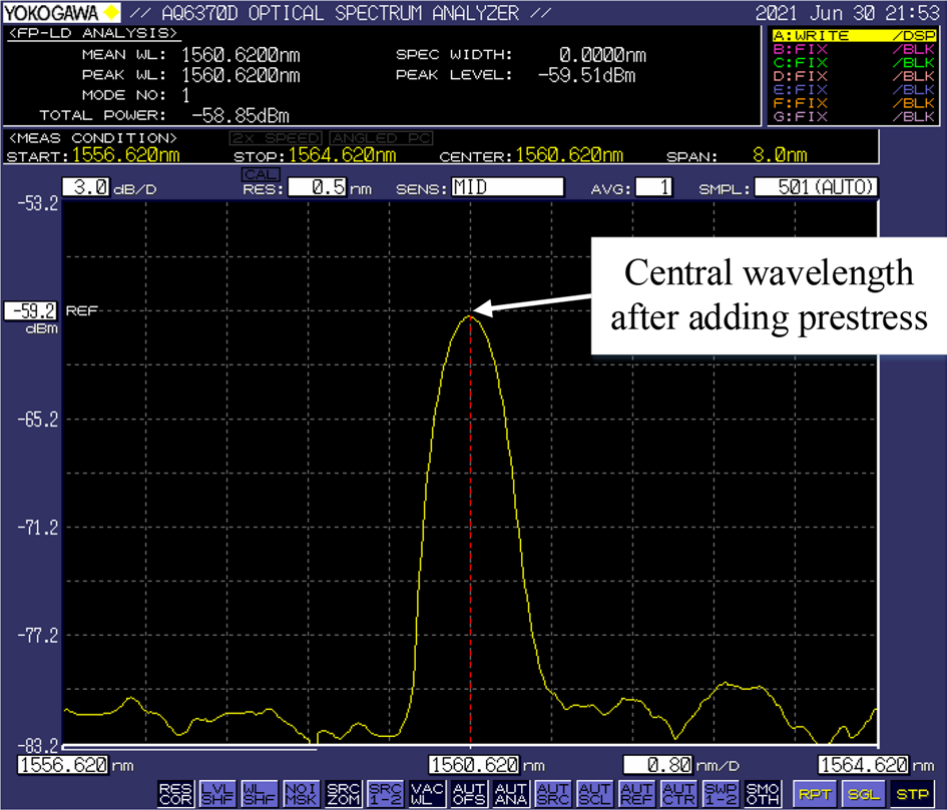
The spectrum of the FBG sensor at room temperature and force-free [the figure was generated by Microsoft Visio Professional 2016 (https://www.microsoft.com/zh-CN/download/details.aspx?id=51188)].

The FBG used in the sensor is three fiber Bragg gratings in the same batch, whose central wavelength is 1560.5 nm, reflectivity is ≥ 90%, and the length of the FBG gate region is 10 mm. The reflection spectrum is shown in Fig. 8.

**Figure 9:**
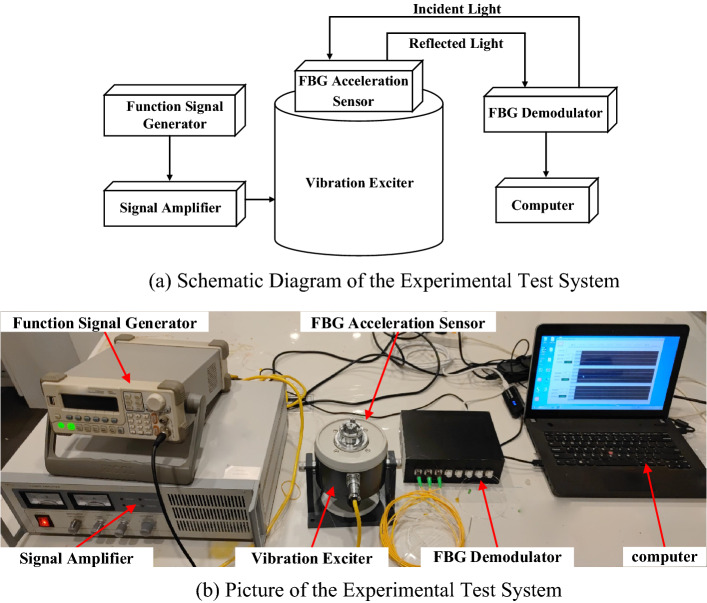
FBG acceleration sensor test system [**(a,b)** were generated by Microsoft Visio Professional 2016 (https://www.microsoft.com/zh-CN/download/details.aspx?id=51188)].

The apparatus for experimental test of the sensor principally included function signal generator, signal amplifier, standard exciter, FBG demodulator, computer, etc. The test was performed with a function signal generator (DG1022) from RIGOL Technologies with a sampling rate of 1 GSa/a, 14 quasi-waveform functions and abundant standard interfaces designed for users to remotely control the data transmission of instruments and USB interfaces via Web. The signal amplifier (MWY-TZQ50) was manufactured by Beijing Weiyun with a frequency response range of 1–15,000 Hz and an SNR of more than 75 dB to work with the function signal generator to amplify the function signals. The FBG demodulator (MWY-FBG-CS800) was also manufactured by Beijing Weiyun with a sampling frequency of up to 1 kHz and a built-in laser source to deliver the transmitted light waves to the multi-cantilever beam low-frequency acceleration sensor on the vibration exciter system via optical fibers; additionally, the FBG demodulator has 8 independent demodulation channels, which can effectively distinguish the reflection spectra of different FBGs, conducted spectral analysis and data acquisition inside, and transferred the acquired data to the computer in the end. The experimental test system established with above-noted devices for the multi-cantilever beam low-frequency FBG acceleration sensor is shown in Fig. 9.

### Analysis of amplitude-frequency response characteristics

**Figure 10:**
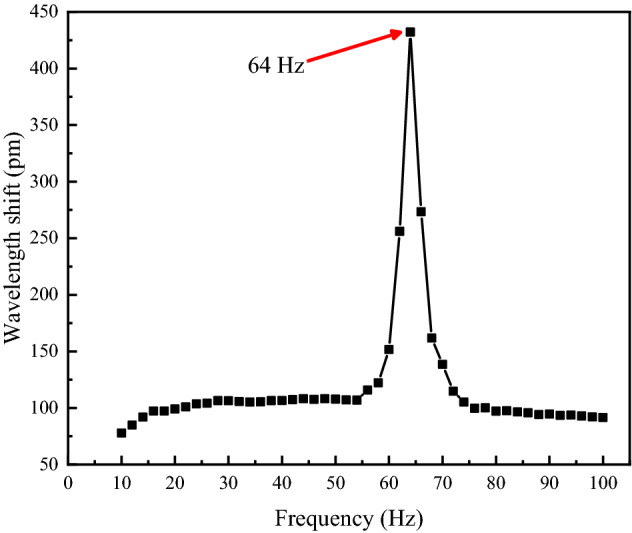
Amplitude-frequency response of the FBG accelerometer [the figure was generated by Origin 2016 × 64 (https://www.originlab.com/2016)].

First, the signal generator was adjusted in the experiment so that the vibration exciter could deliver sinusoidal excitation signals with an acceleration amplitude of $2.5{\text{ m/s}}^{2}$ for the sensor sweep test. According to the simulation result, the sweep frequency range was set to 10–100 Hz at a step of 10 Hz. The approximate range of the natural frequency of sensor was identified, and then the test was repeated at a step of 2 Hz; the ultimate amplitude-frequency response curve of the sensor is shown in Fig. 10.

As shown in Fig. 10, the natural frequency is approximately 64 Hz, and the response was relatively gentle within the frequency range of 16–54 Hz, thus being substantially are applicable for the measurement of low-frequency signals; moreover, the experimental measurement of the natural frequency was close to 68 Hz, a simulation calculated value; the minor errors might result from the mechanical losses that occurred between components of the screwed sensor under external excitations.

### Linear response test

**Figure 11:**
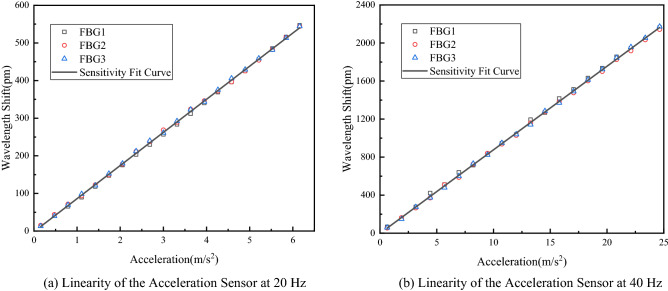
Linearity of the acceleration sensor [**(a,b)** were generated by Origin 2016 × 64 (https://www.originlab.com/2016)].

To study the linearity and sensitivity of the multi-cantilever beam acceleration sensor, the signal generator was so adjusted that the output frequency of vibration exciter was 20 Hz and 40 Hz. Due to the vibration exciter constraints, the variation amplitude of acceleration at the vibration signal frequency of 20 Hz was $0.1{\text{ m/s}}^{ - 2}$-$6.1{\text{ m/s}}^{ - 2}$,at a step of 0.3 ${\text{m/s}}^{ - 2}$. The variation amplitude of acceleration at the vibration signal frequency of 40 Hz was ${0}{\text{.6 m/s}}^{ - 2}$-${24}{\text{.6 m/s}}^{ - 2}$, at a step of ${1}{\text{.2 m/s}}^{ - 2}$. The acceleration response curve plotted is shown in Fig. 11.

As shown in Fig. 11, at 20 Hz, the measuring sensitivity of multi-cantilever beam FBG acceleration sensor is $88.02{\text{ pm/m}}\;{\text{s}}^{ - 2}$, while the fit coefficient R^2^ = 0.9995; at 40 Hz, the measuring sensitivity of multi-cantilever beam FBG acceleration sensor is $87.89{\text{ pm/m}}\;{\text{s}}^{ - 2}$, while the fit coefficient R^2^ = 0.9947; in conclusion, the experiment determined the sensitivity of multi-cantilever beam FBG acceleration sensor to be approximately $87.955\,\,{\text{ pm/m}}\,\,{\text{s}}^{ - 2}$, and determined the natural frequency to be about 64 Hz; hence, it's applicable for detecting violation signals with the frequency range of 16–54 Hz. With the requirements for low-frequency vibration measurement met, it's more sensitive than other FBG acceleration sensors.

In addition to the sensitivity, dynamic range is one of the key parameter indicators of sensor^16^. When it comes to FBG acceleration sensor, the dynamic range $D_{R}$ is associated with maximum wavelength shift $\lambda_{\max }$ and minimum wavelength drift $\lambda_{\min }$ detectable by sensor; the relational expression is

7 $$D_{R} = 20\lg (\frac{{\lambda_{\max } }}{{\lambda_{\min } }})$$

Furthermore, maximum wavelength drift $\lambda_{\max }$ is principally restricted by the elastic deformation range of elastic element of the sensor as well as the grating prestress; minimum wavelength drift $\lambda_{\min }$ is principally dependent on the FBG demodulation system resolution. In the sensor sensitivity test experiment, the maximum wavelength shift from the sensor was $2172{\text{ pm}}$, while the resolution of FBG demodulator for the experiment was $0.1{\text{ pm}}$; the calculated dynamic range of the sensor was up to 86 dB, which substantially met the requirements of ANSS (advanced national seismic system) on dynamic range of the class-C acceleration sensors^17^.

### Transverse interference immunity experiment

**Figure 12:**
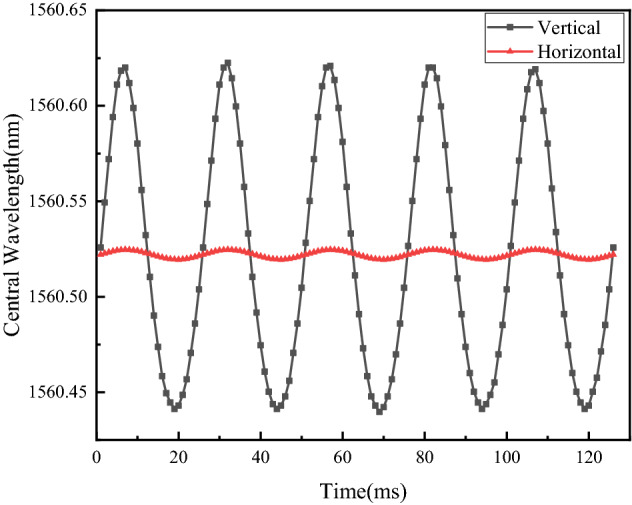
Transverse interference immunity behavior of the sensor [the figure was generated by Origin 2016 × 64 (https://www.originlab.com/2016)].

Transverse interference behavior is also an important performance indicator to consider for single-DOF acceleration sensors. The sensor employs three cantilever beams to clamp the mass block at its center; no matter from which side the transverse interference is applied, the cantilever beam will provide pulling force or supporting force at the same time, thereby alleviating the deformation of fiber grating, so as to reduce the transverse interference. To study the transverse interference immunity behavior of the acceleration sensor, its bottom was fixed to the side of the horizontal vibration exciter during the experiment so that the vibrating direction was perpendicular to the measuring spindle of the sensor. The sensor was brought under an acceleration excitation signal with a frequency of 40 Hz and an amplitude of ${2}{\text{.5 m/s}}^{ - 2}$, and the shift value of output wavelength was compared with respective result in direction of the measuring spindle to plot the characteristic curve of sensor's transverse interference immunity, as shown in Fig. 12.

Figure 12 indicates a transverse wavelength shift of approximately 5.2 pm, a principal wavelength shift of about 201.9 pm, and a transverse interference of less than 2.58%, which demonstrates the favorable transverse interference immunity of sensor.

### Impulse response experiment

**Figure 13:**
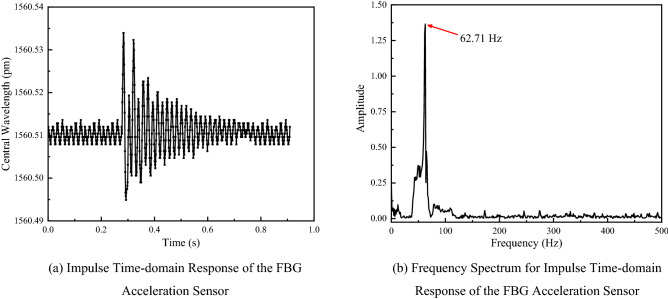
Impulse response behavior of the sensor [**(a,b)** were generated by Origin 2016 × 64 (https://www.originlab.com/2016)].

Compared with excitation signal, impulse signal is an unsteady transient signal that contains abundant vibration information to adequately reflect the response characteristics of acceleration sensor. Along with it, it's possible to verify the natural frequency of the sensor. The generation of an impulse signal was simulated by instantly knocking on the surface of vibration exciter during the experiment; the results of impulse response test and Fourier spectrum analysis within the time domain are shown in Fig. 13.

According to Fig. 13, the multi-cantilever beam FBG acceleration sensor can effectively reflect impulse excitation; the Fourier analysis of the time domain data brings about a frequency spectrum, where the natural frequency of sensor is about 62.71 Hz, which is close to the simulation result and the experimental result of the sensor amplitude frequency; the minor errors may result from the inadequate fixation between vibration exciter surface and sensor; the artificial percussion for impulse signal simulation leads to the resonance between vibration exciter surface and sensor, thereby causing minor errors.

### The repeatability of the sensor measurement

**Figure 14:**
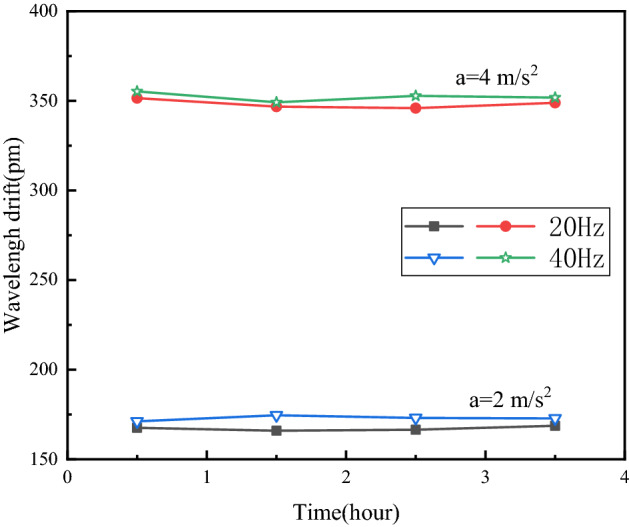
Sensor repeatability test [the figure was generated by Origin 2016 × 64 (https://www.originlab.com/2016)].

The test repeatability data is one of the effective evidences that the sensor can maintain a good working condition for a long time. In this test, the output frequency of the vibration table is set to 20 Hz and 40 Hz, and the acceleration is ${\text{2 m/s}}^{ - 2}$ and ${\text{4 m/s}}^{ - 2}$. Every 60 min, the vibration is suspended for 30 min for 30 min, and the measurement is repeated four times to test the corresponding stability of the sensor output. The test uses the relative standard deviation RSD to represent the repeatability error of the sensor, and its expression is

8 $$RSD{ = }\frac{SD}{{\overline{x}}} \times 100\% = \left( {\frac{{\sqrt {\frac{{\sum\nolimits_{i = 1}^{n} {(x_{i} - \overline{x})^{2} } }}{n - 1}} }}{{\overline{x}}}} \right) \times 100\%$$

where, *SD* is the standard deviation and $\overline{x}$ the corresponding mean value. The experimental results are shown in Fig. 14.

As shown in Fig. 14, when the frequency is 20 Hz, the relative standard deviations of the FBG center wavelength drift corresponding to ${\text{2 m/s}}^{ - 2}$ and ${\text{4 m/s}}^{ - 2}$ are 0.715% and 0.572%, respectively. When the frequency is 40 Hz, the relative standard deviations of the FBG center wavelength drift corresponding to ${\text{2 m/s}}^{ - 2}$ and ${\text{4 m/s}}^{ - 2}$ are 1.216% and 0.871%, respectively. It can be seen that the repeatability error of the sensor is small and the stability is good in the ideal flat area.

## Conclusion

The present paper proposes a multi-cantilever beam low-frequency FBG acceleration sensor, where three cantilever beams are evenly arranged at 120° to clamp the mass in the center, while the sensitivity and transverse interference immunity are improved. Simulation analysis was used with experimental verification to optimize the design of the sensor and perform performance tests. According to the research findings, the sensors, whose natural frequency is approx. 64 Hz, is applicable for monitoring the vibration signals within the range 16–54 Hz. The sensor sensitivity is approximately $87.955{\text{ pm/m}}\quad {\text{s}}^{ - 2}$, the transverse interference immunity being lower than 2.58%, and the dynamic range being up to 86 dB. The sensor designed in the paper is more compact, more sensitive, good stability and more dynamically extensive than other cantilever type FBG acceleration sensors. But there is still much room for improvement; for instance, when used for dynamic monitoring, the sensor may vibrate tens of thousands of times a day; if things go on like this, the fixing screws and cantilever beams among various parts of the sensor may develop fatigue wear, which may in turn impair the sensor performance. Moreover, since the sensor has not yet introduced reference fibers for temperature compensation, it may fail in an operating environment with significant temperature differences. Hence, the original scheme can be further improved so that the sensor can be used for monitoring researches in water conservancy & electric power, bridges & dams, engineering seismology, etc.
